# Supplementary material for: Systematic review and meta-analysis of the factors affecting waning of post-vaccination neutralizing antibody responses against SARS-CoV-2
Source: NPJ Vaccines. 2023 Oct 20;8:159. doi: 10.1038/s41541-023-00756-1 (PMC10589259; doi:10.1038/s41541-023-00756-1)
Supplement: Supplementary file 1 — Supplementary material [file 41541_2023_756_MOESM1_ESM.pdf]

Supplementary material

Supplementary figure 1: Reliability assessment

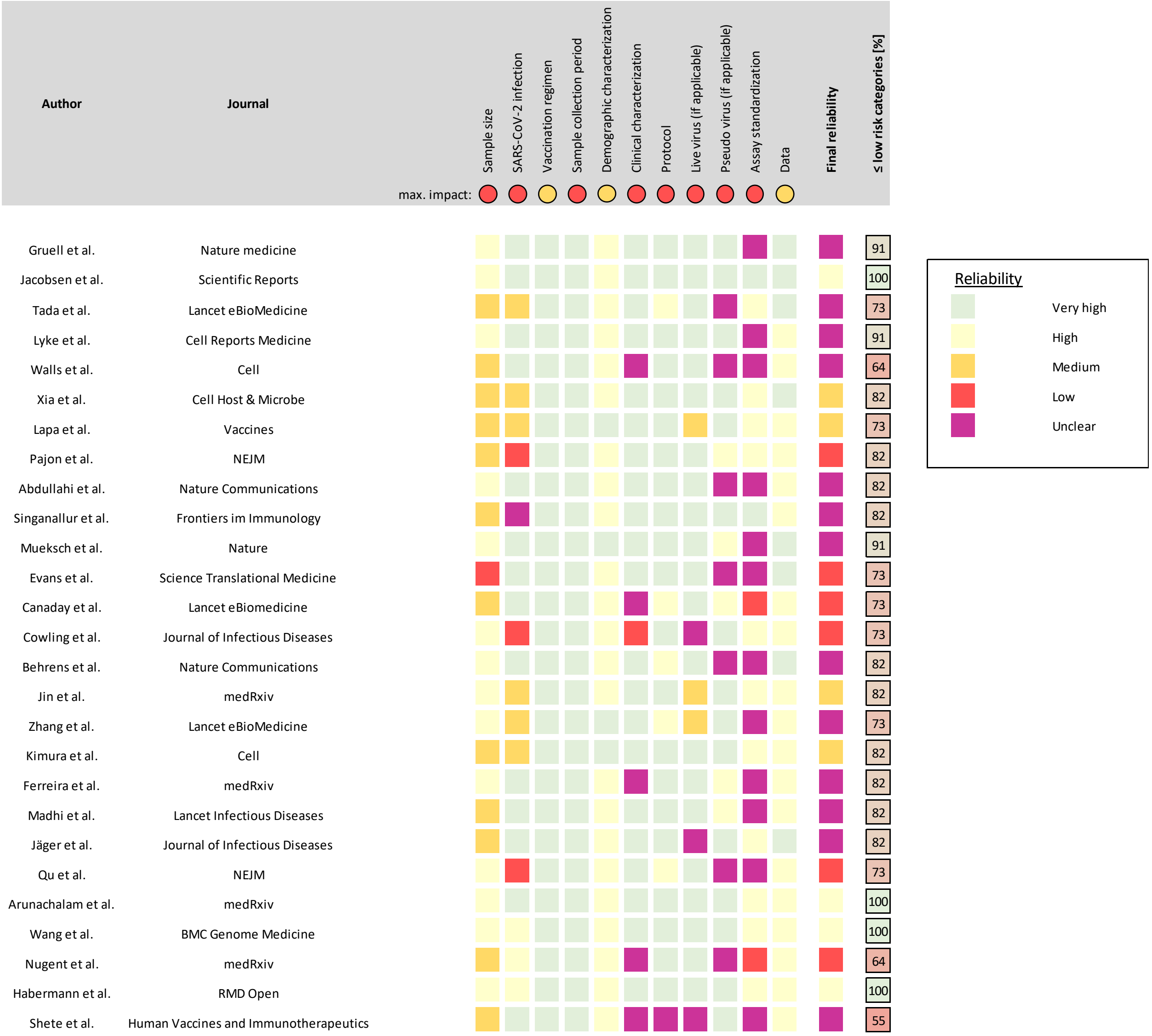

Supplementary Figure 1: Reliability assessment. All studies that were included in the meta-analyses were assessed with a reliability score using a previously published tool[5]. Studies were considered to have high reliability if no criterion had more than a low risk score (yellow), medium reliability if no criterion was above a medium risk score (orange) and low if at least one criterion met a high risk score (red). No study met the “very high” reliability score (no criterion with risk of bias). Studies with at least one criterion that could not be assessed (e.g. no data provided or unclear), received an unclear reliability (purple). Eleven categories are assessed by the tool and assigned an independent risk score. The maximum impact a category can have is shown as “max impact” indicating the worst possible outcome for this category. The percentage of categories with a low or no risk is shown on the right, complementing the final reliability.

<u>Supplementary figure 2: Reliability assessment, sensitivity analysis</u>

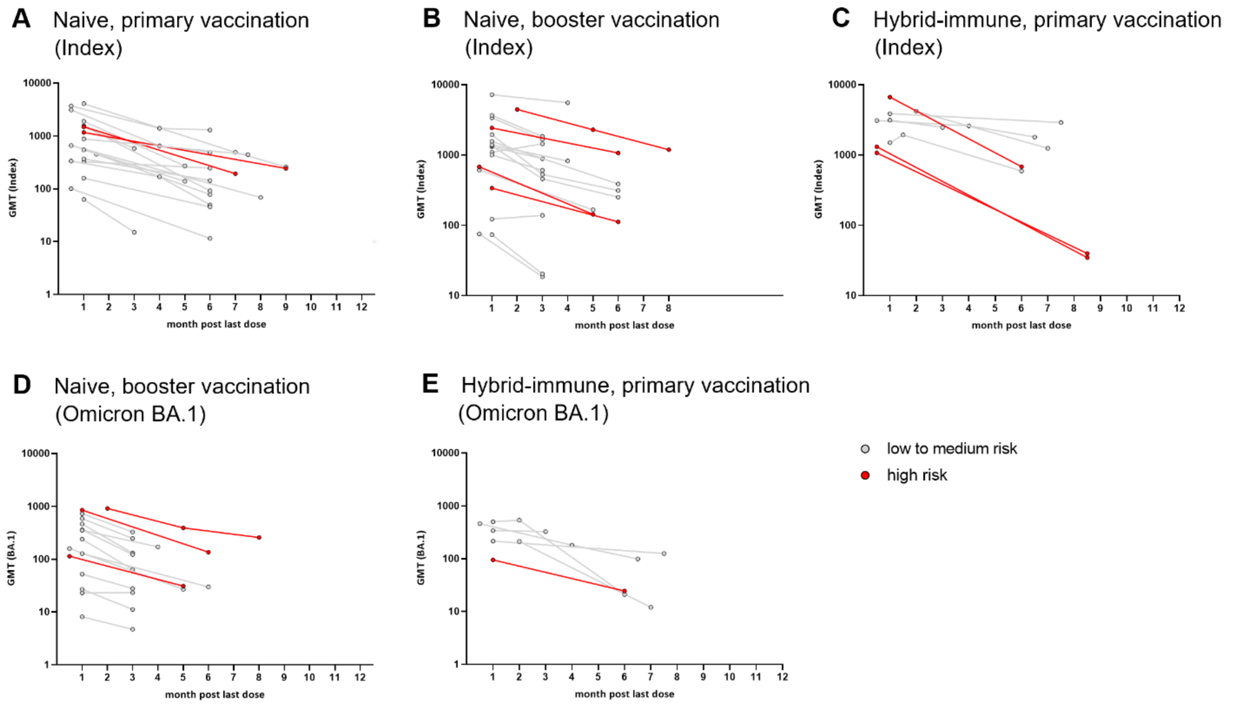

Supplementary Figure 2: Reliability assessment of included studies. Rates of waning against the Index strain (A – C) and against Omicron BA.1 (D and E) shown colored according to reliability as assessed by a previously published standardized reliability assessment tool[5]. Studies assessed as high to medium reliability are shown in grey, low reliability studies are shown in red. Studies with unclear reliability are included to “low to medium risk”. Abbreviations: GMT, geometric mean titer; Index denotes SARS-CoV-2 Wuhan-like including D614G-strains.

Supplementary Appendix 1: Cohort characteristics and abstracted data.

ID	Study First Author	Study population	Naive										Hybrid					
			Primary series					Boost vaccination					Primary series					
			Vaccine	Number of doses	Time since final dose (months)	WT GMT (95% CI)	BA.1 GMT (95% CI)	Vaccine	Number of doses	Time since final dose (months)	WT GMT (95% CI)	BA.1 GMT (95% CI)	Vaccine	Number of doses	Infecting strain	Time since final dose (months)	WT GMT (95% CI)	BA.1 GMT (95% CI)
24	Jacobsen	General	BNT+BNT	2	1	159.0 (89.3 - 283.0)*												
			BNT+BNT	2	6	45.4 (27.2 - 75.8)*												
46	Tada	General	BNT+BNT	2	1	878.2 (562.1 - 1371.9)*							BNT+BNT	2	n/a	1	3868.0 (2730.7 - 5479.0)*	215.3 (188.1 - 246.4)*
			BNT+BNT	2	7.5	443.7 (277.0 - 710.8)*							BNT+BNT	2	n/a	7.5	2903.8 (1960.0 - 4302.2)*	125.0 (108.0 - 144.6)*
63	Lyke	General						BNT+BNT+J&J	3	1	1090.5 (807.4 - 1473.0)	358.9 (250.0 - 515.4)						
								BNT+BNT+J&J	3	3	1444.3 (906.2 - 2302.0)	123.0 (81.8 - 185.0)						
								BNT+BNT+BNT	3	1	1305.5 (1044.3 - 1631.9)	464.1 (341.3 - 631.1)						
								BNT+BNT+BNT	3	3	886.1 (643.5 - 1220.1)	131.0 (91.5 - 487.6)						
								MOD+MOD+MOD	3	1	3383.0 (2673.3 - 4281.2)	586.8 (426.0 - 808.4)						
								MOD+MOD+MOD	3	3	1668.0 (1049.0 - 2652.4)	247.1 (170.3 - 358.4)						
								J&J+BNT	2	1	1001.6 (784.6 - 1278.7)	238.8 (173.9 - 327.9)						
								J&J+BNT	2	3	606.0 (387.5 - 947.6)	63.6 (46.1 - 87.8)						
								J&J+J&J	2	1	122.8 (92.0 - 163.8)	26.9 (19.5 - 37.2)						
								J&J+J&J	2	3	138.2 (89.3 - 213.9)	11.2 (8.8 - 14.2)						
68	Walls	General	RNA+RNA	2	0.5	3700.0 (n/a)							RNA+RNA	2	n/a	0.5	3100.0 (n/a)	460.0 (n/a)
			RNA+RNA	2	4	1400.0 (n/a)							RNA+RNA	2	n/a	4	2600.0 (n/a)	180.0 (n/a)
			RNA+RNA	2	6	1300.0 (n/a)							RNA+RNA	2	n/a	6.5	1800.0 (n/a)	99.0 (n/a)
70	Xia	General	BNT+BNT	2	0.5	660.5 (466.7 - 934.8)*		BNT+BNT+BNT	3	1	1390.0 (956.0 - 2021.6)*	353.3 (252.2 - 495.0)*						
			BNT+BNT	2	8	68.3 (39.7 - 117.4)*		BNT+BNT+BNT	3	4	819.9 (574.2 - 1170.8)*	170.9 (113.6 - 257.2)*						
127	Pajon	General	MOD+MOD	2	1	1496.0 (916.3 - 2457.3)		MOD+MOD+MOD	3	1	2423.0 (1543.5 - 3750.2)	850.0 (459.4 - 1610.1)						
			MOD+MOD	2	7	193.0 (118.8 - 318.5)		MOD+MOD+MOD	3	6	1067.0 (752.3 - 1543.5)	136.0 (72.5 - 257.8)						
			MOD+MOD	2	1	1165.0 (n/a)												
			MOD+MOD	2	9	242.5 (n/a)												
149	Singanallur	General	BNT+BNT	2	0.5	100.8 (26.3 - 175.3)												
			BNT+BNT	2	6	11.5 (8.3 - 14.6)												
152	Muecksch	General	RNA+RNA	2	1	1892.8 (1405.0 - 2550.0)*												
			RNA+RNA	2	5	272.2 (190.8 - 388.4)*												
217	Cowling	General						INA+INA+BNT	3	1	338.0 (n/a)							
								INA+INA+BNT	3	6	112.0 (n/a)							

ID	Study First Author	Study population	Naive										Hybrid					
			Primary series					Boost vaccination					Primary series					
			Vaccine	Number of doses	Time since final dose (months)	WT GMT (95% CI)	BA.1 GMT (95% CI)	Vaccine	Number of doses	Time since final dose (months)	WT GMT (95% CI)	BA.1 GMT (95% CI)	Vaccine	Number of doses	Infecting strain	Time since final dose (months)	WT GMT (95% CI)	BA.1 GMT (95% CI)
228	Behrens	HCW	BNT+BNT	2	1	4096.5 (3032.2 - 5534.3)*												
			BNT+BNT	2	7	490.8 (337.5 - 713.7)*												
			BNT+BNT	2	9	259.6 (190.1 - 354.4)*												
			VAX+VAX	2	0.5	335.8 (224.3 - 502.8)*												
			VAX+VAX	2	4	169.3 (119.6 - 239.7)*												
			VAX+VAX	2	6	49.6 (30.3 - 81.2)*												
			VAX+BNT	2	0.5	3104.7 (2425.2 - 3974.6)*												
			VAX+BNT	2	4	650.2 (535.7 - 789.2)*												
			VAX+BNT	2	6	490.4 (359.7 - 668.6)*												
232	Jin	General						COV+COV+Ad5_low	3	1	1937.3 (1466.9 - 2558.4)	52.0 (37.2 - 72.6)						
								COV+COV+Ad5_low	3	3	530.1 (412.5 - 681.1)	27.9 (18.8 - 41.3)						
								COV+COV+Ad5_low	3	6	312.9 (237.7 - 411.8)							
								COV+COV+Ad5_hi	3	1	1350.8 (952.6 - 1915.3)	23.1 (15.7 - 33.9)						
								COV+COV+Ad5_hi	3	3	457.6 (349.4 - 599.2)	23.3 (16.2 - 33.3)						
								COV+COV+Ad5_hi	3	6	251.1 (178.2 - 354.0)							
								COV+COV+COV	3	1	73.5 (52.3 - 103.3)							
								COV+COV+COV	3	3	20.4 (14.3 - 29.1)							
272	Zhang	General						COV+COV+COV	3	0.5	75.4 (61.4 - 92.5)	8.1 (6.1 - 10.7)						
								COV+COV+COV	3	3	18.5 (14.9 - 22.9)	4.7 (4.1 - 5.5)						
274	Kimura	General						BNT+BNT+BNT	3	1	7210.0 (6340.0 - 8200.0)							
								BNT+BNT+BNT	3	4	5530.0 (4330.0 - 6850.0)							
277	Ferreira	General	VAX+VAX	2	1	368.9 (201.2 - 676.5)*												
			VAX+VAX	2	6	143.6 (83.8 - 246.1)*												
284	Madhi	General	VAX+VAX	2	1.5	451.0 (197.0 - 1035.0)							VAX+VAX	2	WT	1	1496.0 (768.0 - 2916.0)	499.0 (282.0 - 885.0)
			VAX+VAX	2	6	78.0 (19.0 - 329.0)							VAX+VAX	2	WT	1.5	1933.0 (1283.0 - 2912.0)	535.0 (290.0 - 988.0)
													VAX+VAX	2	WT	6	590.0 (337.0 - 1032.0)	21.0 (14.0 - 32.0)
292	Jäger	General	VAX+VAC	2	1	325.4 (234.8 - 450.9)												
			VAX+VAC	2	6	246.3 (192.7 - 314.6)												
130	Abdullahi	General	VAX+VAX	2	1	1495.9 (582.2 - 3843.3)*							VAX+VAX	2	Pre-Omicron	1	3127.6 (1969.4 - 4967.0)*	340.8 (239.8 - 484.2)*
			VAX+VAX	2	3	581.8 (217.3 - 1557.6)*							VAX+VAX	2	Pre-Omicron	3	2471.4 (1617.3 - 3776.5)*	325.7 (235.1 - 451.2)*

ID	Study First Author	Study population	Naive										Hybrid					
			Primary series					Boost vaccination					Primary series					
			Vaccine	Number of doses	Time since final dose (months)	WT GMT (95% CI)	BA.1 GMT (95% CI)	Vaccine	Number of doses	Time since final dose (months)	WT GMT (95% CI)	BA.1 GMT (95% CI)	Vaccine	Number of doses	Infecting strain	Time since final dose (months)	WT GMT (95% CI)	BA.1 GMT (95% CI)
153	Evans	HCW	RNA+RNA	2	1	1558.2 (1159.8 - 2093.4)*							RNA+RNA	2	Pre-Omicron	1	6680.4 (2832.7 - 15754.7)*	94.7 (13.1 - 682.2)*
			RNA+RNA	2	6	91.4 (56.7 - 147.3)*							RNA+RNA	2	Pre-Omicron	6	680.7 (152.6 - 3037.1)*	24.4 (3.1 - 191.8)*
219	Qu	HCW						RNA+RNA+RNA	3	2	4448.0 (n/a)	917.0 (n/a)						
								RNA+RNA+RNA	3	5	2285.0 (n/a)	392.0 (n/a)						
								RNA+RNA+RNA	3	8	1187.0 (n/a)	257.0 (n/a)						
321	Arunachalam	General						RNA+RNA+RNA	3	1	1557.3 (1527.2 - 1587.4)*	127.6 (116.1 - 139.1)*						
								RNA+RNA+RNA	3	6	386.2 (365.0 - 407.4)*	29.9 (23.0 - 36.8)*						
344	Wang	General	BBIBP+BBIBP	2	1	63.0 (n/a)												
			BBIBP+BBIBP	2	3	15.0 (n/a)												
365	Nugent	HCW						RNA+RNA+RNA	3	0.5	680.0 (467.0 - 990.0)	114.0 (61.0 - 211.0)						
								RNA+RNA+RNA	3	5	143.0 (82.0 - 251.0)	31.0 (17.0 - 56.0)						
		Older adults						RNA+RNA+RNA	3	0.5	611.0 (404.0 - 925.0)	159.0 (99.0 - 256.0)						
								RNA+RNA+RNA	3	5	166.0 (77.0 - 174.0)	27.0 (19.0 - 40.0)						
176	Habermann	General						VAC+VAC+BNT	3	1	3661.0 (1556.8 - 8404.0)	729.0 (270.1 - 1889.1)						
								VAC+VAC+BNT	3	3	1842.0 (919.2 - 3523.0)	329.0 (129.4 - 814.6)						
162	Canaday	HCW											BNT+BNT	2	Pre-Omicron	0.5	1073.0 (475.0 - 2426.0)	
													BNT+BNT	2	Pre-Omicron	8.5	39.6 (17.7 - 88.3)	
		Older adults											BNT+BNT	2	Pre-Omicron	0.5	1311.0 (697.0 - 2469.0)	
													BNT+BNT	2	Pre-Omicron	8.5	34.6 (19.2 - 62.1)	
285	Shete	n/a											VAX+VAX	2	WT	2	4190.0 (3218.0 - 5542.0)	212.0 (75.8 - 623.0)
													VAX+VAX	2	WT	7	1248.0 (713.0 - 2287.0)	12.0 (1.6 - 97.0)
14	Gruell	General	BNT+BNT	2	1	546.3 (382.9 - 779.5)*												
			BNT+BNT	2	5	138.6 (27.2 - 75.8)*												

Supplementary Appendix 2: Study grading.

		Reference	Tada et al.		Lyke et al.		Walls et al.		Xia et al.		Lapa et al.		Pajon et al.		Abdullahi et al.		Gilboa et al.		Singanallur et al.		Mueksch et al.	
**Category**	**Aspect**	**Parameter / explanation**	**Status**	**Impact on reliability**	**Status**	**Impact on reliability**	**Status**	**Impact on reliability**	**Status**	**Impact on reliability**	**Status**	**Impact on reliability**	**Status**	**Impact on reliability**	**Status**	**Impact on reliability**	**Status**	**Impact on reliability**	**Status**	**Impact on reliability**	**Status**	**Impact on reliability**
Cohort details	Sample size	**Sample size**<br>*How many samples were included?*	5-20		21-50		5-20		5-20		5-20		5-20		21-50		21-50		5-20		21-50	
	SARS-CoV-2 infection	**Reported**<br>*Was pre-vaccination COVID-19 considered?*	Yes - and subjects stratified		Yes - only naive included		Yes - and subjects stratified		Not reported and early pandemic setting		Yes - and subjects stratified		Yes - only naive included		Yes - and subjects stratified		Yes - only naive included		Not reported and late pandemic setting		Yes - only naive included	
		**Confirmed**<br>*Previous infection confirmed by NP-ELISA or similar means?*	Yes		Yes		Yes		No / not reported		Yes		Yes		Yes		Yes		N. a.		Yes	
		**Breakthrough cases reported**<br>*Are breakthrough cases reported? Applicable if this is relevant in the context of the study.*	No		Yes		Yes		No		No		Yes		Yes		Yes		N. a.		Yes	
		**Breakthrough cases stratified**<br>*If breakthrough cases occured, did the authors stratify?*	N. a.		Yes		Yes		N. a.		N. a.		No		Yes		Yes		N. a.		N. a.	
	Vaccination regimen	**Dosing interval reported**<br>*Do the authors report the dosing interval (if applicable)?*	N. a.		Yes		N. a.		Yes		N. a.		Yes		N. a.		Yes		N. a.		N. a.	
		**Stratified by partial / full immunization**<br>*Do the authors stratiy by partial and full immunization?*	N. a.		N. a.		N. a.		N. a.		N. a.		N. a.		N. a.		N. a.		N. a.		N. a.	
	Sample collection period	**≥7 days post last dose**<br>*Were all samples taken at least seven days post final dose?*<br>*Adjust to "YES" if <10% of samples are taken earlier.*	Yes		Yes		Yes		Yes		Yes		Yes		Yes		Yes		Yes		Yes	
		**Stratified OR 14 d - 4 mo post full immunization**<br>*Are the results stratified OR are all samples taken between 2 weeks and 4 months post final dose?*<br>*Adjust to "YES" if <20% of sample do not comply.*	Yes		Yes		Yes		Yes		Yes		Yes		Yes		Yes		Yes		Yes	
	Demographic characterization	**Age distribution reported**<br>*Is the age distribution (range) of all subjects reported?*	Yes		Yes		Yes		Yes		Yes		Yes		No		Yes		Yes		Yes	
		**Stratified by age group (<18 or 18 - 59 or ≥60)**<br>*Adjust to "YES" if <20% of samples belong to different age groups*	Yes		No		No		No		Yes		No		No		No		Yes		Yes	
		**Sex distribution reported**<br>*Is the sex distribution of all subjects reported?*	Yes		Yes		Yes		Yes		Yes		Yes		No		Yes		Yes		Yes	
		**Stratified by sex <u>or</u> equal sex distribution**<br>*Equal sex distribution: 50% ±10% per sex.*<br>*Adjust to "YES" if <20% of samples were not stratified.*	Equal sex distribution		Equal sex distribution		Not stratified nor equally distributed		Not stratified nor equally distributed		Equal sex distribution		Equal sex distribution		Not stratified nor equally distributed		Not stratified nor equally distributed		Not stratified nor equally distributed		Equal sex distribution	
		**Cohort selection unbiased**<br>*If only a subgroup of the initial study cohort was analyzed, did the cohort selection happen unbiased (no pre-selection of high-titre reponders etc)?*	Yes		Yes		Yes		Yes		Yes		Yes		Yes		Yes		Yes		Yes	
		**Study period and geographic location reported**<br>*Applicable if SARS-CoV-2 infections occured and variant identification & distribution is not reported for the cohort.*	Yes		N. a.		Yes		Yes		N. a.		N. a.		Yes		N. a.		N. a.		N. a.	
		**Variant prevalence reported**<br>*Applicable if any SARS-CoV-2 infections occured.*	Yes		N. a.		Yes		N. a.		N. a.		N. a.		Yes		N. a.		N. a.		N. a.	
		**Stratified by variant prevalence**<br>*Applicable if SARS-CoV-2 infections with multiple variants occured.*	No		N. a.		Yes		N. a.		N. a.		N. a.		No		N. a.		N. a.		N. a.	
	Clinical characterization	**Reported**<br>*Is any relevant clinical characterization reported?*<br>*Applicable if at least one third of the study cohort is likely or known to have clinical conditions that might affect immunity.*	Yes		N. a.		No		N. a.		N. a.		N. a.		N. a.		N. a.		N. a.		N. a.	
		**Stratifed by immunocompromised**<br>*Applicable if clinical characterization applies.*<br>*Adjust to "YES" if <20% of samples were not stratified.*	Yes		N. a.		N. a.		N. a.		N. a.		N. a.		N. a.		N. a.		N. a.		N. a.	
Assay details	Protocol	**Assay type reported**<br>*Is the precise assay type and endpoint reported (pseudovirus vs life virus, NT20, NT50, NT80 etc)?*	Yes		Yes		Yes		Yes		Yes		Yes		Yes		Yes				Yes	
		**Precise protocol reported**<br>*Do the authors provide a precise protocol for the neutralization assay within the manuscript?*	No		Yes		Yes		Yes		Yes		Yes		Yes		Yes		Yes		Yes	
	Live virus strain (if applicable)	**Virus lineage reported**<br>*Applicable if live virus neutralization was performed.*	N. a.		N. a.		N. a.		Yes		Yes		Yes		N. a.		No		Yes		N. a.	
		**Sequence confirmation by sequencing**<br>*Applicable if live virus neutralization was performed.*	N. a.		N. a.		N. a.		Yes		No		Yes		N. a.		No		Yes		N. a.	
	Pseudo virus strain (if applicable)	**Construct details reported**<br>*Applicable if pseudovirus neutralization was performed.*	No		Yes		No		N. a.		N. a.		Yes		No		N. a.		N. a.		Yes	
		**All variant-associated spike mutations**<br>*Applicable if pseudovirus neutralization was performed.*<br>*Are all variant-<u>associated</u> spike mutations included to the pseudovirus?*	N. a.		Yes		No		N. a.		N. a.		Yes		N. a.		N. a.		N. a.		Yes	
		**Sequence confirmation by sequencing**<br>*Applicable if pseudovirus neutralization was performed.*	N. a.		Yes		No		N. a.		N. a.		No		N. a.		N. a.		N. a.		No	
	Assay standardization	**Virus titre reported and consistent**<br>*Are virus titres used for neutralization assays reported and if so: consistent and with small input variance?*	Consistent and with small variance		Not reported		Not reported		Consistent and with small variance		Consistent and with small variance		Consistent and with small variance		Not reported		Consistent and with small variance		Consistent and with small variance		Not reported	
		**Error in titre reported by back titration**<br>*Was the virus titre used for neutralization assays confirmed by the authors by back-titration or similar means?*	No		No		No		No		No		No		No		No		Yes		No	
		**WHO IS antibody used**<br>*WHO international standard antibody used for standardization?*	No																			
		**Details on cell culture reported**<br>*Are precise details on cell culture reported*<br>*(cell culture conditions, maximum passage number etc)?*	Yes		Yes		Yes		Yes		Yes		No		No		No		Yes			
Data	Data reporting	**Raw data reported**<br>*Raw data for neutralization titres reported?*	Yes		No		No		Yes		No		No		No		No		No		Yes	
		**Reference virus is appropriate (non VoI/VoC)**<br>*Is the reference virus used for calculating variant-specific fold-changes reasonable (non VoI/VoC)?*	Yes		Yes		Yes		Yes		Yes		Yes		Yes		Yes		Yes		Yes	
		**Data shown as individual values with statistics**<br>*Are individual data points and apropriate statistics provided?*	Yes		Yes		Yes		Yes		Yes		Yes		Yes		Yes		Yes		Yes	
			**Overall risk of low reliability:**	UNCLEAR	**Overall risk of low reliability:**	UNCLEAR	**Overall risk of low reliability:**	UNCLEAR	**Overall risk of low reliability:**	MEDIUM	**Overall risk of low reliability:**	MEDIUM	**Overall risk of low reliability:**	HIGH	**Overall risk of low reliability:**	UNCLEAR	**Overall risk of low reliability:**	UNCLEAR	**Overall risk of low reliability:**	UNCLEAR	**Overall risk of low reliability:**	UNCLEAR

		Reference	Evans et al.		Canaday et al.		Cowling et al.		Behrens et al.		Jin et al.		Zhang et al.		Kimura et al.		Ferreira et al.		Madhi et al.		Jaeger et al.	
**Category**	**Aspect**	**Parameter / explanation**	**Status**	**Impact on reliability**	**Status**	**Impact on reliability**	**Status**	**Impact on reliability**	**Status**	**Impact on reliability**	**Status**	**Impact on reliability**	**Status**	**Impact on reliability**	**Status**	**Impact on reliability**	**Status**	**Impact on reliability**	**Status**	**Impact on reliability**	**Status**	**Impact on reliability**
Cohort details	Sample size	**Sample size**<br>*How many samples were included?*	<5		5-20		21-50		21-50		21-50		21-50		5-20		21-50		5-20		5-20	
	SARS-CoV-2 infection	**Reported**<br>*Was pre-vaccination COVID-19 considered?*	Yes - and subjects stratified		Yes - and subjects stratified		Yes - only naive included		Yes - only naive included		Yes - only naive included		Yes - only naive included		Yes - only naive included		Yes - only naive included		Yes - and subjects stratified		Yes - only naive included	
		**Confirmed**<br>*Previous infection confirmed by NP-ELISA or similar means?*	Yes		Yes		No / not reported		Yes		No / not reported		No / not reported		No / not reported		Yes		Yes		Yes	
		**Breakthrough cases reported**<br>*Are breakthrough cases reported? Applicable if this is relevant in the context of the study.*	Yes		Yes		No		Yes		No		No		No		Yes		Yes		Yes	
		**Breakthrough cases stratified**<br>*If breakthrough cases occured, did the authors stratify?*	Yes		Yes		No		Yes		N. a.		N. a.		N. a.		N. a.		Yes		N. a.	
	Vaccination regimen	**Dosing interval reported**<br>*Do the authors report the dosing interval (if applicable)?*	N. a.		N. a.		Yes		Yes		Yes		Yes		N. a.		N. a.		N. a.		N. a.	
		**Stratified by partial / full immunization**<br>*Do the authors stratiy by partial and full immunization?*	N. a.		Yes		Yes		Yes		N. a.		Yes		Yes		Yes		Yes		Yes	
	Sample collection period	**≥7 days post last dose**<br>*Were all samples taken at least seven days post final dose?*<br>*Adjust to "YES" if <10% of samples are taken earlier.*	Yes		Yes		Yes		Yes		Yes		Yes		Yes		Yes		Yes		Yes	
		**Stratified OR 14 d - 4 mo post full immunization**<br>*Are the results stratified OR are all samples taken between 2 weeks and 4 months post final dose?*<br>*Adjust to "YES" if <20% of sample do not comply.*	Yes		Yes		Yes		Yes		Yes		Yes		Yes		Yes		Yes		Yes	
	Demographic characterization	**Age distribution reported**<br>*Is the age distribution (range) of all subjects reported?*	Yes		Yes		Yes		Yes		Yes		Yes		Yes		Yes		Yes		Yes	
		**Stratified by age group (<18 or 18 - 59 or ≥60)**<br>*Adjust to "YES" if <20% of samples belong to different age groups*	Yes		Yes		No		Yes		Yes		Yes		Yes		Yes		Yes		Yes	
		**Sex distribution reported**<br>*Is the sex distribution of all subjects reported?*	Yes		Yes		Yes		Yes		Yes		Yes		Yes		Yes		Yes		Yes	
		**Stratified by sex <u>or</u> equal sex distribution**<br>*Equal sex distribution: 50% ±10% per sex.*<br>*Adjust to "YES" if <20% of samples were not stratified.*	Equal sex distribution		Not stratified nor equally distributed		Not stratified nor equally distributed		Not stratified nor equally distributed		Not stratified nor equally distributed		Equal sex distribution		Equal sex distribution		Not stratified nor equally distributed		Equal sex distribution		Not stratified nor equally distributed	
		**Cohort selection unbiased**<br>*If only a subgroup of the initial study cohort was analyzed, did the cohort selection happen unbiased (no pre-selection of high-titre reponders etc)?*	Yes		Yes		Yes		Yes		Yes		Yes		Yes		Yes		Yes		Yes	
		**Study period and geographic location reported**<br>*Applicable if SARS-CoV-2 infections occured and variant identification & distribution is not reported for the cohort.*	Yes		Yes		N. a.		N. a.		N. a.		N. a.		N. a.		N. a.		Yes		N. a.	
		**Variant prevalence reported**<br>*Applicable if any SARS-CoV-2 infections occured.*	No		No		N. a.		N. a.		N. a.		N. a.		N. a.		N. a.		No		N. a.	
		**Stratified by variant prevalence**<br>*Applicable if SARS-CoV-2 infections with multiple variants occured.*	No		No		N. a.		N. a.		N. a.		N. a.		N. a.		N. a.		No			
	Clinical characterization	**Reported**<br>*Is any relevant clinical characterization reported?*<br>*Applicable if at least one third of the study cohort is likely or known to have clinical conditions that might affect immunity.*	N. a.		No		Yes		N. a.		N. a.		N. a.		N. a.		No		N. a.		N. a.	
		**Stratifed by immunocompromised**<br>*Applicable if clinical characterization applies.*<br>*Adjust to "YES" if <20% of samples were not stratified.*	N. a.				No		N. a.		N. a.		N. a.		N. a.		N. a.		N. a.		N. a.	
Assay details	Protocol	**Assay type reported**<br>*Is the precise assay type and endpoint reported (pseudovirus vs life virus, NT20, NT50, NT80 etc)?*	Yes		Yes		Yes		Yes		Yes		Yes		Yes		Yes		Yes		Yes	
		**Precise protocol reported**<br>*Do the authors provide a precise protocol for the neutralization assay within the manuscript?*	Yes		No		Yes		No		Yes		No		Yes		Yes		Yes		Yes	
	Live virus strain (if applicable)	**Virus lineage reported**<br>*Applicable if live virus neutralization was performed.*	N. a.		N. a.		No		N. a.		Yes		Yes		N. a.		N. a.		N. a.		No	
		**Sequence confirmation by sequencing**<br>*Applicable if live virus neutralization was performed.*	N. a.		N. a.		No		N. a.		No		No		N. a.		N. a.		N. a.		N. a.	
	Pseudo virus strain (if applicable)	**Construct details reported**<br>*Applicable if pseudovirus neutralization was performed.*	No		Yes		N. a.		No		N. a.		N. a.		Yes		Yes		Yes		N. a.	
		**All variant-associated spike mutations**<br>*Applicable if pseudovirus neutralization was performed.*<br>*Are all variant-<u>associated</u> spike mutations included to the pseudovirus?*	N. a.		Yes		N. a.		N. a.		N. a.		N. a.		N. a.		N. a.		Yes		N. a.	
		**Sequence confirmation by sequencing**<br>*Applicable if pseudovirus neutralization was performed.*	N. a.		No		N. a.		No		N. a.		N. a.		Yes		No		No		N. a.	
	Assay standardization	**Virus titre reported and consistent**<br>*Are virus titres used for neutralization assays reported and if so: consistent and with small input variance?*	Not reported		Not consistent or with high variance		Consistent and with small variance		Not reported		Consistent and with small variance		Not reported		Consistent and with small variance		Not reported		Not reported		Consistent and with small variance	
		**Error in titre reported by back titration**<br>*Was the virus titre used for neutralization assays confirmed by the authors by back-titration or similar means?*	No		No		No		No		No		No		No		No		No		No	
		**WHO IS antibody used**<br>*WHO international standard antibody used for standardization?*																				
		**Details on cell culture reported**<br>*Are precise details on cell culture reported*<br>*(cell culture conditions, maximum passage number etc)?*	Yes		No		No		No		No		No		Yes		No		No		Yes	
Data	Data reporting	**Raw data reported**<br>*Raw data for neutralization titres reported?*	Yes		No		No		Yes		No		No		No		No		No		Yes	
		**Reference virus is appropriate (non VoI/VoC)**<br>*Is the reference virus used for calculating variant-specific fold-changes reasonable (non VoI/VoC)?*	Yes		Yes		Yes		Yes		Yes		Yes		Yes		Yes		Yes		Yes	
		**Data shown as individual values with statistics**<br>*Are individual data points and apropriate statistics provided?*	Yes		Yes		No		Yes		Yes		Yes		Yes		Yes		Yes		Yes	
			**Overall risk of low reliability:**	HIGH	**Overall risk of low reliability:**	HIGH	**Overall risk of low reliability:**	HIGH	**Overall risk of low reliability:**	UNCLEAR	**Overall risk of low reliability:**	MEDIUM	**Overall risk of low reliability:**	UNCLEAR	**Overall risk of low reliability:**	MEDIUM	**Overall risk of low reliability:**	UNCLEAR	**Overall risk of low reliability:**	UNCLEAR	**Overall risk of low reliability:**	UNCLEAR

		Reference	Qu et al.		Arunachalam et al.		Wang et al.		Nugent et al.		Habermann et al.		Shete et al.		Gruell et al.		Jacobsen et al.	
**Category**	**Aspect**	**Parameter / explanation**	**Status**	**Impact on reliability**	**Status**	**Impact on reliability**	**Status**	**Impact on reliability**	**Status**	**Impact on reliability**	**Status**	**Impact on reliability**	**Status**	**Impact on reliability**	**Status**	**Impact on reliability**	**Status**	**Impact on reliability**
Cohort details	Sample size	**Sample size** *How many samples were included?*	21-50		21-50		21-50		5-20		21-50		5-20		21-50		21-50	
	SARS-CoV-2 infection	**Reported** *Was pre-vaccination COVID-19 considered?*	Yes - and subjects stratified		Yes - and subjects stratified		Yes - and subjects stratified		Yes - only naive included		Yes - only naive included		Yes - and subjects stratified		Yes - only naive included		Yes - only naive included	
		**Confirmed** *Previous infection confirmed by NP-ELISA or similar means?*	No / not reported		Yes		No / not reported		No / not reported		No / not reported		Yes		Yes		Yes	
		**Breakthrough cases reported** *Are breakthrough cases reported? Applicable if this is relevant in the context of the study.*	Yes		Yes		Yes		Yes		Yes		Yes		N. a.		Yes	
		**Breakthrough cases stratified** *If breakthrough cases occured, did the authors stratify?*	No		N. a.		N. a.		N. a.		Yes		Yes		N. a.		N. a.	
	Vaccination regimen	**Dosing interval reported** *Do the authors report the dosing interval (if applicable)?*	N. a.		Yes		Yes		N. a.		Yes		N. a.		N. a.		Yes	
		**Stratified by partial / full immunization** *Do the authors stratiy by partial and full immunization?*	Yes		Yes		Yes		Yes		Yes		N. a.		N. a.		Yes	
	Sample collection period	**≥7 days post last dose** *Were all samples taken at least seven days post final dose? Adjust to "YES" if <10% of samples are taken earlier.*	Yes		Yes		Yes		Yes		Yes		Yes		Yes		Yes	
		**Stratified OR 14 d - 4 mo post full immunization** *Are the results stratified OR are all samples taken between 2 weeks and 4 months post final dose? Adjust to "YES" if <20% of sample do not comply.*	Yes		Yes		Yes		Yes		Yes		Yes		Yes		Yes	
	Demographic characterization	**Age distribution reported** *Is the age distribution (range) of all subjects reported?*	Yes		Yes		Yes		Yes		Yes		No		Yes		Yes	
		**Stratified by age group (<18 or 18 - 59 or ≥60)** *Adjust to "YES" if <20% of samples belong to different age groups*	Yes		No		Yes		No		No		Not reported		No		Yes	
		**Sex distribution reported** *Is the sex distribution of all subjects reported?*	Yes		Yes		Yes		Yes		Yes		No		Yes		Yes	
		**Stratified by sex or equal sex distribution** *Equal sex distribution: 50% ±10% per sex. Adjust to "YES" if <20% of samples were not stratified.*	Not stratified nor equally distributed		Not stratified nor equally distributed		Not stratified nor equally distributed		Stratified		Not stratified nor equally distributed		Not stratified nor equally distributed		Equal sex distribution		Not stratified nor equally distributed	
		**Cohort selection unbiased** *If only a subgroup of the initial study cohort was analyzed, did the cohort selection happen unbiased (no pre-selection of high-titre reponders etc)?*	Yes		Yes		Yes		Yes		Yes		Yes		Yes		Yes	
		**Study period and geographic location reported** *Applicable if SARS-CoV-2 infections occured and variant identification & distribution is not reported for the cohort.*	Yes		N. a.		N. a.		N. a.		N. a.		Yes		N. a.		N. a.	
		**Variant prevalence reported** *Applicable if any SARS-CoV-2 infections occured.*	Yes		N. a.		N. a.		N. a.		N. a.		Yes		N. a.		N. a.	
		**Stratified by variant prevalence** *Applicable if SARS-CoV-2 infections with multiple variants occured.*	No		N. a.		N. a.		N. a.		N. a.		No		N. a.		N. a.	
	Clinical characterization	**Reported** *Is any relevant clinical characterization reported? Applicable if at least one third of the study cohort is likely or known to have clinical conditions that might affect immunity.*	N. a.		N. a.		N. a.		No		Yes		No		Yes		Yes	
		**Stratifed by immunocompromised** *Applicable if clinical characterization applies. Adjust to "YES" if <20% of samples were not stratified.*	N. a.		N. a.		N. a.		N. a.		Yes		N. a.		Yes		Yes	
Assay details	Protocol	**Assay type reported** *Is the precise assay type and endpoint reported (pseudovirus vs life virus, NT20, NT50, NT80 etc)?*	Yes		Yes		Yes		Yes		Yes		No		Yes		Yes	
		**Precise protocol reported** *Do the authors provide a precise protocol for the neutralization assay within the manuscript?*	No		Yes		Yes		Yes		Yes		No		Yes		Yes	
	Live virus strain (if applicable)	**Virus lineage reported** *Applicable if live virus neutralization was performed.*	N. a.		Yes		N. a.		N. a.		N. a.		No		N. a.		N. a.	
		**Sequence confirmation by sequencing** *Applicable if live virus neutralization was performed.*	N. a.		Yes		N. a.		N. a.		N. a.		N. a.		N. a.		N. a.	
	Pseudo virus strain (if applicable)	**Construct details reported** *Applicable if pseudovirus neutralization was performed.*	No		N. a.		Yes		No		Yes		N. a.		Yes		Yes	
		**All variant-associated spike mutations** *Applicable if pseudovirus neutralization was performed. Are all variant-associated spike mutations included to the pseudovirus?*	N. a.		N. a.		Yes		N. a.		Yes		N. a.		Yes		Yes	
		**Sequence confirmation by sequencing** *Applicable if pseudovirus neutralization was performed.*	No		N. a.		Yes		N. a.		Yes		N. a.		Yes		Yes	
	Assay standardization	**Virus titre reported and consistent** *Are virus titres used for neutralization assays reported and if so: consistent and with small input variance?*	Not reported		Consistent and with small variance		Consistent and with small variance		Not consistent or with high variance		Consistent and with small variance		Not reported		Not reported		Consistent and with small variance	
		**Error in titre reported by back titration** *Was the virus titre used for neutralization assays confirmed by the authors by back-titration or similar means?*	No		No		No		No		No		No		No		Yes	
		**WHO IS antibody used** *WHO international standard antibody used for standardization?*																
		**Details on cell culture reported** *Are precise details on cell culture reported (cell culture conditions, maximum passage number etc)?*	No		Yes		Yes		No		No		No		Yes		Yes	
Data	Data reporting	**Raw data reported** *Raw data for neutralization titres reported?*	No		No		No		No		No		No		Yes		Yes	
		**Reference virus is appropriate (non VoI/VoC)** *Is the reference virus used for calculating variant-specific fold-changes reasonable (non VoI/VoC)?*	Yes		Yes		Yes		Yes		Yes		Yes		Yes		Yes	
		**Data shown as individual values with statistics** *Are individual data points and apropriate statistics provided?*	Yes		Yes		No		Yes		Yes		Yes		Yes		Yes	
			**Overall risk of low reliability:**	HIGH	**Overall risk of low reliability:**	LOW	**Overall risk of low reliability:**	LOW	**Overall risk of low reliability:**	HIGH	**Overall risk of low reliability:**	LOW	**Overall risk of low reliability:**	UNCLEAR	**Overall risk of low reliability:**	UNCLEAR	**Overall risk of low reliability:**	LOW
